# Anatomical and functional outcomes of combined ventral rectopexy and sacrocolpo/hysteropexy for multicompartment pelvic organ prolapse: a systematic review and meta-analysis

**DOI:** 10.1007/s10151-025-03236-x

**Published:** 2025-12-08

**Authors:** Alessandro Ferdinando Ruffolo, Tomaso Melocchi, Chrystèle Rubod, Yohan Kerbage, Giuseppe Campagna, Sara Mastrovito, Alfredo Ercoli, Giovanni Panico, Michel Cosson, Marine Lallemant

**Affiliations:** 1 CHU Lille, Service de Chirurgie Gynécologique, 59000 Lille, France; 2 Department of Gynecology, Fondazione IRCCS San Gerardo Dei Tintori, Monza, Italy; 3 Faculté de Médecine, University of Lille, 59000 Lille, France; 4 University of Lille, CNRS, Centrale Lille, UMR 9013—LaMcube—Laboratoire de Mécanique, Multiphysique, Multiéchelle, 59000 Lille, France; 5 Univiversity of Lille, Unité Inserm U1189-OncoThai: Laser Assisted Therapies and Immunotherapies for Oncology, 59000 Lille, France; 6 UOC of Gynecological Surgery and Urogynecology, Centre of Excellence Women and Childbirth, Ospedale Isola Tiberina—Gemelli Isola, Rome, Italy; 7 Department of Woman and Child’s Health and Public Health, Division of Urogynecology and Reconstructive Surgery of Pelvic Floor, Fondazione Policlinico Universitario Agostino Gemelli IRCCS, Rome, Italy; 8 Department of Human Pathology in Adult and Childhood “G. Barresi”, Unit of Gynecology and Obstetrics, University of Messina, Messina, Italy; 9 University of Lille, CHU Lille, ULR 2694—METRICS, 59000 Lille, France

**Keywords:** Ventral rectopexy, Sacrocolpopexy, Sacrohysteropexy, Pelvic organ prolapse, Rectal prolapse, Obstructed defecation syndrome, Anal incontinence

## Abstract

**Introduction:**

Limited data exists in literature regarding concomitant ventral rectopexy (VRP) and sacrocolpo/hysteropexy (SCP/SHP), with existing studies being predominantly retrospective. The aim of this meta-analysis is to assess the anatomical and functional outcomes of combined VRP and SCP/SHP for the treatment of multicompartmental pelvic organ prolapse (POP).

**Methods:**

We performed systematic research and meta-analysis from PubMed/MEDLINE and EMBASE, according to Preferred Reporting Items for Systematic Reviews and Meta-Analyses (PRISMA) 2020 guidelines, until 15 January 2025. Women submitted to VRP with SCP/SHP were included. Improvement of anorectal symptoms were evaluated. Postoperative anatomical relapse was reported. Re-operation rates were evaluated.

**Results:**

Six articles were included. Constipation/obstructed defecation syndrome (ODS) [odds ratio (OR) 0.26, 95% CI 0.10–0.68; *p* = 0.006 (*I*^2^ test 81%, *p* = 0.56)] and of anal/fecal incontinence (AI/FI) rates [OR 0.09, 95% CI 0.03–0.30; *p* < 0.0001 (*I*^2^ test 70%, *p* = 0.04)] significantly improved after combined VRP and SCP/SHP. The proportion metanalysis of four included studies reported a subjective POP recurrence rate of 7% (95% CI 1–13%; *I*^2^ test 82.9%, *p* < 0.001). The proportion metanalysis of five included studies for objective POP recurrence was 5% (95% CI 1–9%; *I*^2^ test 56.9%, *p* = 0.041). No serious adverse events were reported.

**Conclusions:**

VRP combined with SCP/SHP has been shown to be safe and effective for women with multicompartment POP, providing optimal anatomical and functional outcomes. Larger, long-term, prospective-controlled studies are needed to confirm these results.

## Introduction

Pelvic organ prolapse (POP) is the descent of the anterior, posterior, and/or apical vaginal compartments with protrusion of pelvic organs into the vagina [1]. It is caused by anatomical distortion of the integrated support of the pelvic floor provided by the muscles, nerves, and connective tissue attachments such as uterosacral-cardinal ligaments and endopelvic fascia. The prevalence of POP in the female population is around 24% and tends to increase with age [2]. Symptoms of pelvic floor dysfunction, such as urinary incontinence, sexual dysfunction, and obstructed defecation syndrome (ODS) or anal/fecal incontinence (AI/FI), are often seen simultaneously, as the defect frequently coexists in the urogenital and rectal compartments [3, 4], and can reduce quality of life (QoL). Surgical treatment is required for 11–20% of cases and up to 30% of patients may require a re-operation [5, 6].

Defects involving the posterior pelvic compartment can lead to posterior pelvic floor disorders. Anatomically, these include the rectocele, peritoneocele/enterocele, and rectal prolapse. These anatomical conditions can be associated with functional anorectal symptoms such as ODS and AI/FI. Depending on the severity of symptoms and imaging findings, surgical repair may be necessary. Various approaches to surgical treatment of posterior pelvic floor disorders have been described, including transvaginal rectocele repair, abdominal sacrocolpopexy, stapled transanal rectal resection (STARR), and ventral rectopexy [7, 8]. For anterior and/or apical POP, sacrocolpopexy remains one of the most preferred surgical approaches [9, 10].

As mentioned above, in 10–55% of patients with multicompartmental prolapse, there is a deterioration of pelvic floor integrity [11, 12]. Therefore, surgeons are currently inclined to perform sacrocolpopexy and rectopexy procedures simultaneously to correct multicompartmental prolapse in a multidisciplinary setting [13]. This combined approach is associated with significant improvements in long-term surgical and anatomical outcomes and self-reported symptoms [14, 15].

Previously described by D’Hoore, laparoscopic ventral mesh rectopexy avoids posterolateral dissection of the rectum. The similarity of the pelvic dissections performed during sacrocolpopexy and ventral rectopexy allows these two procedures to be safely performed simultaneously [16]. Minimally invasive techniques have been preferred for prolapse repair owing to shorter hospital stays and faster recovery. Favorable results and improved urinary, anorectal, and prolapse symptoms have been reported following laparoscopic sacrocolpo–rectopexy with synthetic mesh [17].

Given the paucity of robust data in literature regarding concomitant ventral rectopexy and sacrocolpopexy, with existing studies being predominantly retrospective, we conducted a systematic review to analyze the available evidence and synthesize more robust scientific evidence regarding the combined surgical approach. The aim of this meta-analysis was to assess the anatomical and functional outcomes of combined ventral rectopexy and sacrocolpo/hysteropexy for the treatment of multicompartmental pelvic organ prolapse.

## Methods

This study was performed according to the Preferred Reporting Items for Systematic Reviews and Meta-Analyses (PRISMA) 2020 guidelines [18]. The protocol was registered in PROSPERO (CRD42025638155).

### Eligibility criteria

We included the available studies that evaluated the anatomical and functional outcomes of combined ventral rectopexy (VRP) and sacrcolpo/hysteropexy (SCP/SHP). Only studies evaluating endoscopic (laparoscopic or robotic) VRP in combination with SCP/SHP were included. No studies using suture rectopexy were included. The following study designs were considered appropriate: randomized controlled trials (RCTs) and observational prospective or retrospective cohort studies. We excluded review articles, case series, case reports, commentaries, editorials, meeting abstracts, and non-English articles. There were no restrictions on the year of publication.

### Information source

The systematic literature search was performed using the PubMed, Medical Literature Analysis and Retrieval System Online (MEDLINE), and EMBASE databases (last search date: 15 January 2025).

### Search strategy

The terms used in combination for the literature search were:

For PubMed research:

(“sacrocolpopexies” [All Fields] OR “sacrocolpopexy” [All Fields] OR “sacrohysteropexy” [All Fields]) AND (“rectopexies” [All Fields] OR “rectopexy” [All Fields]).

For Embase research:

(‘sacrocolpopexy’/exp OR sacrocolpopexy OR’sacrohysteropexy’/exp OR sacrohysteropexy) AND (‘rectopexy’/exp OR rectopexy).

All relevant articles were carefully evaluated, and their reference lists were examined to identify other manuscripts that could be retrieved in this review.

### Selection process

Two independent reviewers (A.F.R. and T.M.) selected each article for inclusion by title and abstract, and excluded unrelated studies. Disagreements were resolved by consensus, including a third author (M.C.), who reviewed the eligible studies. Potential eligible studies were assessed in full to decide whether to include them.

### Data collection

Structured tables were used to extract the necessary data from each eligible study. Data extracted included: authors’ names, year of publication, country, study design, sample size, age of patients, inclusion study criteria, surgical indication, surgical technique, previous and concomitant hysterectomy, preoperative POP stage and/or POP compartment, type of posterior compartment defect, assessment of posterior compartment defect, preoperative and postoperative constipation/ODS rate, preoperative and postoperative AI/FI rate, subjective assessment of anorectal symptoms, postoperative POP stage/recurrence (defined as a POP of any compartment ≥ II stage), postoperative bulge symptoms, postoperative rectal prolapse (RP), follow-up, postoperative complications (serious adverse events were considered as Clavien–Dindo stage ≥ IV), and re-operation rate.

### Data items

- Anatomical POP recurrence, defined as the presence of postoperative POP of any compartment ≥ II stage according to the POP-Q system.
- Subjective POP recurrence, defined as the presence of postoperative bulging symptoms.
- Postoperative anorectal symptoms, defined as the presence of constipation/ODS and/or AI/FI. Functional outcomes were extracted as reported in the original studies. The quantitative analysis was based on the percentages of patients with constipation/ODS or AI/FI, as defined by each study

### Study risk of bias assessment

Risk of bias and quality assessment of the included studies were determined for nonrandomized studies using the risk of bias in non-randomized studies of intervention (ROBINS-I) tool [19].

### Statistical analysis

Categorical variables were expressed as absolute and relative (percentage) values. Proportional meta-analysis was performed for several outcome measures. Forest plots were used to graphically present the estimated results. A random effects model [20] was used for the statistical pooling of data. Results were reported as pooled percentages with related 95% confidence intervals (95% CI). Forest plots were used to present odds ratios (ORs) and 95% CIs for the analyzed outcomes. Heterogeneity between studies was assessed using the Higgins *I*^2^-index [21], with *I*^2^ > 50% being considered to indicate the presence of heterogeneity [21, 22]. Meta-analysis was performed using Review Manager version 5.4.1 software (Cochrane Training, London, UK) and Jamovi software version 2.3.28.0 (Sydney, Australia).

## Results

### Study selection

**Fig. 1:**
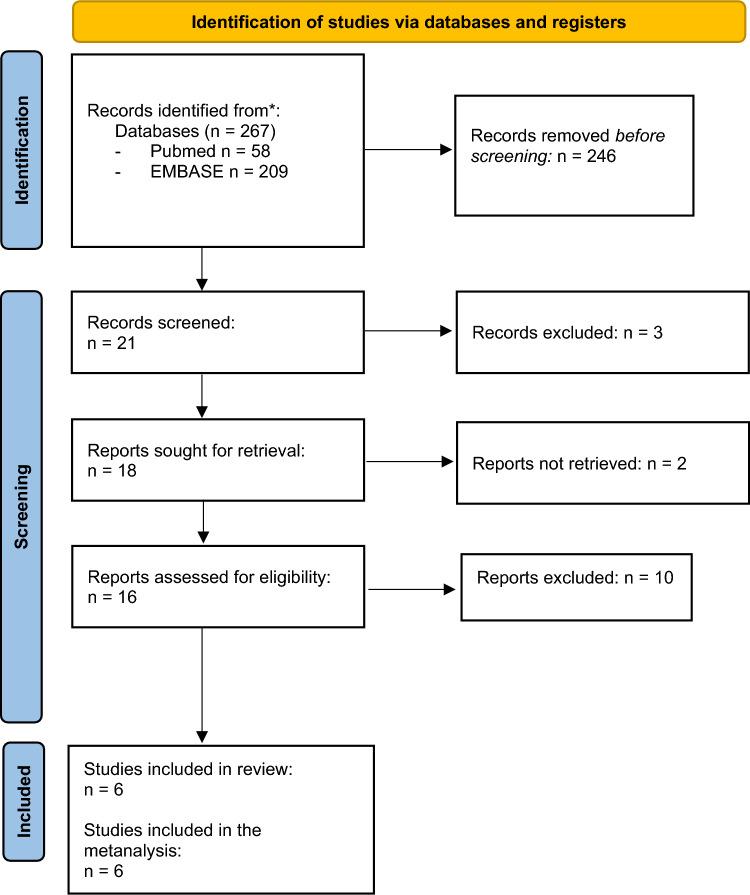
Flow diagram of evidence acquisition in the systematic review and meta-analysis

We identified 209 (EMBASE) and 58 (PubMed) studies (Fig. 1). After checking for duplicate results and titles, 21 articles were selected for abstract screening and 16 for text evaluation. Of these, six studies were selected for inclusion in the systematic review [23–28].

### Risk of bias in studies and levels of evidence

**Fig. 2:**
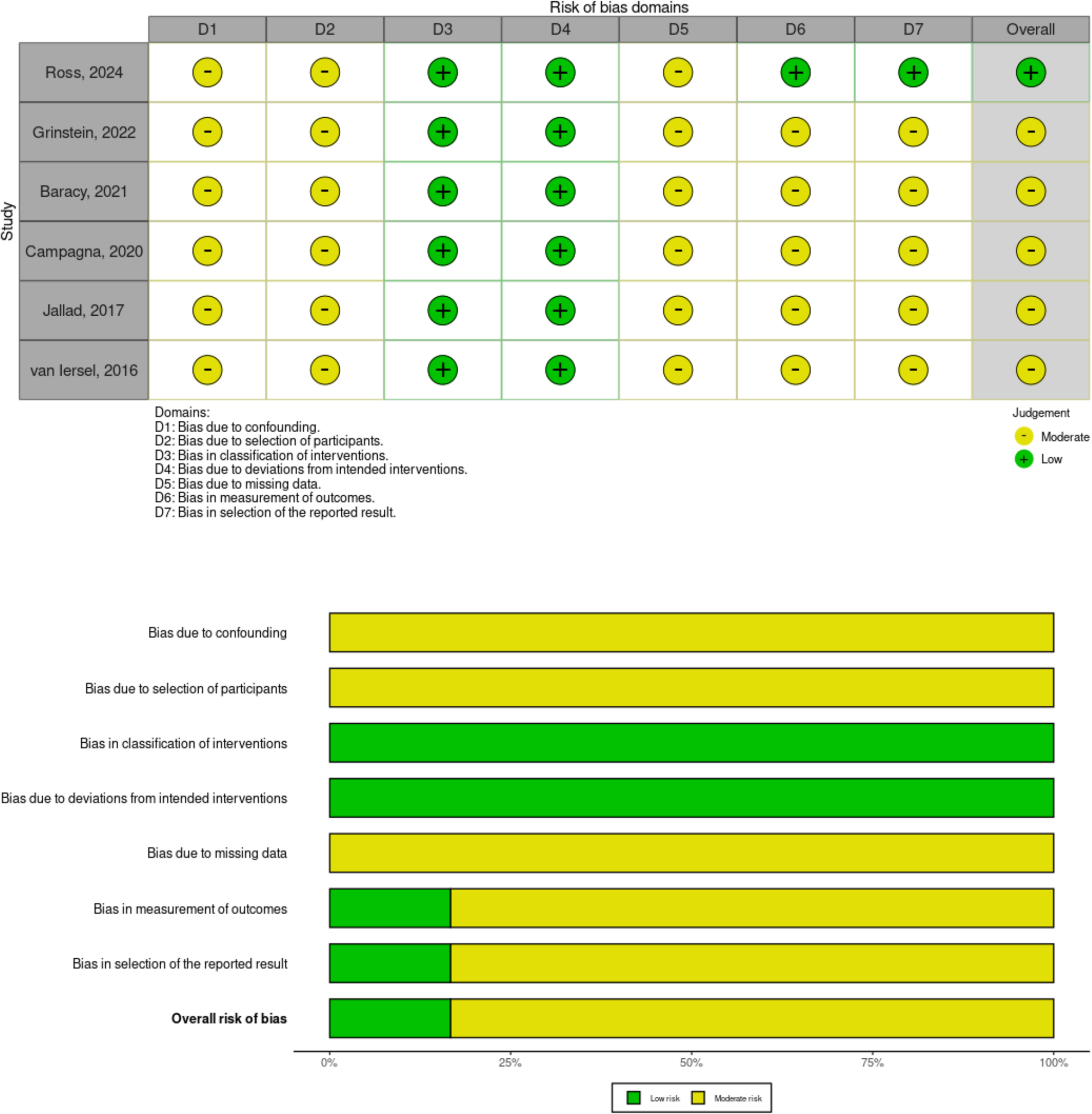
Risk of bias analysis of the six nonrandomized trials

Risk of bias analysis was performed for each study ranging from moderate to low (Fig. 2).

### Study characteristics and study results

Six studies, including 401 women who underwent combined VRP and SCP/SHP for multicompartment POP, were included. Inclusion criteria, surgical indications, and surgical procedure are shown in Table 1. Study characteristics are reported in Table 2. Three studies were conducted in the USA, one study in France, one study in Italy, and one study in the Netherlands. The design was retrospective in five studies and prospective in one study. The mean sample size of women who underwent a VRP and SCP/SHP was 66.8 ± 21.3 patients. The mean and median ages are reported in Table 2. Defecography was used as the main diagnostic test for preoperative assessment of posterior compartment defects. Combined VRP and SCP/SHP postoperative outcomes are reported in Table 3. Mean follow-up ranged from 3 to 36 months.

**Table 1:** Inclusion criteria, surgical indications, and surgical procedures of the included studies

Authors and year	Inclusion criteria	Surgical indication	Surgical technique
Ross 2024	Patients who underwent surgical repair of combined POP and RP/I	POP and RP	R-SCP with R-VRP Biologic or synthetic graft
Grinstein 2022	Patients who underwent L-SHP or L-SCP alone or with concomitant L-VRP	L-SHP or L-SCP for symptomatic POP (POP-Q stage II–IV) L-VRP for external or occult RP on physical examination or imaging, with symptoms of AI or ODS	L-SHP or L-SCP alone or with concomitant L-VRP: a mesh was fixed on the anterior vaginal wall. If posterior compartment was involved, the recto-vaginal space was dissected and an additional mesh was fixed on the posterior vaginal wall. The meshes were sutured to the vagina with 2–0 nonabsorbable polyester material (MersutureTM, Ethicon®). Meshes were fixed on the anterior common vertebral ligament at the level of the promontory with 0 nonabsorbable polyester material (MersutureTM, Ethicon®) in addition to 2–3 Absorba Tack. For L-VRP the recto vaginal space dissection was extended on the posterior vaginal wall over a distance of 10 cm in a vertical plane while respecting the hypogastric and lower rectal nerves The mesh was fixed on the anterior face of the rectum with 2–0 nonabsorbable polyester material (MersutureTM, Ethicon®) Type of mesh: large pore size (≥ 1 mm) lightweight (19 g/m^2^) monofilament polypropylene type-1 prostheses (Coloplast® Group, Restorelle Implant) cut to a width of 4–5 cm and a length of 12–14 cm
Baracy 2021	Women ≥ 18 years underwent R-SCP ± R-VRP	R-SCP for POP R-VRP in case of patients with posterior wall prolapse on POP-Q or fecal incontinence with RP at MR-defecography	R-SCP ± R-VRP: if no previous hysterectomy, a robotic assisted total hysterectomy was performed. A Y-shaped lightweight polypropylene mesh (Vertessa® Lite, Caldara Medical, Augora Hills, CA, USA) was used for the sacrocolpopexy. A 0-delayed-absorbable polydioxanone suture (PDS) in a running fashion was used for the anterior and posterior arms of the vaginal mesh. The vaginal cuff (where applicable) was closed with 0-PDS suture in a running fashion. The mesh was affixed to the sacral promontory with a 0-braided polyester (TiCron, Covidien, Minneapolis, MN, USA), nonabsorbable suture via two simple interrupted sutures
Campagna 2020	Patients treated with L-SCP + L-VRP	Women with III/IV POP-Q stage anterior and/or apical compartment and important rectocele or clinically/defecography-proven rectal prolapse	L-SCP + L-VRP: an adequately shaped macroporous (pore-size of 1 or 3 mm) monofilament, polypropylene mesh (Timesh® PFM medical) is placed and fixed with six 3–0 nonabsorbable sutures (Ethibond Excel® Polyester Suture) to the anterior side of the rectum on its seromuscular layer. A second synthetic mesh fitted to cover the dissected triangular-shaped vesico-vaginal space is inserted and fixed to the anterior vaginal wall with five 3–0 nonabsorbable sutures (Ethibond Excel® Polyester Suture). Three more sutures are placed centrally, in the dorsal part of the uterine cervix. The first mesh is now threaded up toward the promontory, and it is fixed to the longitudinal vertebral ligament previously exposed with one size 0 nonabsorbable suture. The anterior mesh is then threaded up towards the promontory; another size 0 nonabsorbable suture is used for sacral promontory fixation of the cervicovaginal mesh. Supracervical hysterectomy in all patients. Re-peritonealization of the pouch of Douglas using absorbable sutures
Jallad 2017	Patients treated with L/R/O-SHP/SCP + L/R/O-VRP	POP + RP	L/R/O-SHP/SCP + L/R/O-VRP: for the VRP a porcine submucosal intestinal graft (Surgisis Biodesign, Cook Medical, Lafayette, IN) or a lightweight polypropylene mesh graft (NovaSilk or Restorelle, Coloplast, Minneapolis, MN) was used. The graft was placed over the rectum and secured with 10 to 12 delayed absorbable sutures to the rectum, levator ani, and pubococcygeus muscle For SHP/SCP a lightweight polypropylene mesh graft (NovaSilk or Restorelle, Coloplast, Minneapolis, MN) was attached to the vagina with 4–6 delayed absorbable sutures Fixation to the anterior longitudinal ligament of the sacrum (level S1–S2) with two permanent or delayed absorbable sutures without over tensioning. Peritonization with delayed absorbable suture or barbed suture V-lock (Covidien, Minneapolis, MN)
van Iersel 2016	Patients undergoing R-SCP + R-VRP for multicompartment pelvic floor prolapse	Combination of prolapse of the posterior and the middle/anterior compartment, accompanied by a disabling multicompartment problem (i.e., FI, ODS, sensation of prolapse, dyspareunia, and micturition symptoms)	R-SCP + R-VRP: no posterolateral rectal mobilization or lateral ligament dissection was performed. The mesh (Prolene, Ethicon Inc, Johnson & Johnson, Hamburg, Germany; weight 80–85 g/m^2^) was distally attached by sutures (Ethibond, Ethicon Inc) to the ventral aspect of the rectum and proximally to the sacral promontory using titanium tacks (Autosuture Protack 5 mm, Covidien, Mansfield, MA). The mesh was sutured to the posterior vaginal wall. Another mesh was attached to the anterior vaginal wall and cervix with nonabsorbable sutures. Both meshes were then connected with nonabsorbable sutures to the vaginal top or cervix to create a Y-shaped suspension. Peritonization with a 23-cm V-loc suture (Covidien). If the uterus was in situ, a supravaginal hysterectomy was performed

*FI* fecal incontinence, *L* laparoscopic, *POP* pelvic organ prolapse, *POP-Q* pelvic organ prolapse quantification, *O* open, *ODS* obstructed defecation symptoms, *R* robotic, *RP* rectal prolapse, *SCP* sacrocolpopexy, *SHP* sacrohysteropexy, *VRP* ventralrectopexy

**Table 2:** Main characteristics of the studies incorporated in the systematic review

Authors and year	Country	Study design	Sample size, *n*	Age at surgery, years, median or mean (IQR or SD)	Previous hysterectomy, *n* (%)	Preoperative POP stage/type of POP, *n* (%)	Type of posterior compartment defect, *n* (%)	Posterior compartment defect assessment	Preoperative constipation/obstructed defecation, *n* (%)	Preoperative anal/fecal incontinence, *n* (%)	Subjective assessment of anorectal symptoms
Ross, 2024	USA	Retrospective	67	64.1 ± 10.7	36 (53.7)	Anterior Stage II 38/63 (60.3) Stage III/IV 9/63 (14.3) Apical Stage II 13/63 (20.6) Stage III/IV 6/63 (9.5) Posterior Stage II 43/63 (69.4) Stage III/IV 8/63 (13.0)	Rectal prolapse 45 (67.2) Enterocele/sigmoidocele 41 (61.2) Rectocele 39 (58.2)	Defecography	NR	NR	PFDI 122.0 ± 55.1
Grinstein, 2022	France	Retrospective	85	SHP + VRP: 60.2 ± 14 SCP + VRP: 64.5 ± 12.2	18 (21.2)	≥ II	Rectal prolapse 85 (100)	Physical examination or imaging (MR/defecography)	38 (44.7)	64 (75.3)	Wexner incontinence SHP + VRP 7.5 ± 5.3 SCP + VRP 7.2 ± 5.6 PFDI SHP + VRP 158.1 ± 44.9 SCP + VRP 155.9 ± 58
Baracy, 2021	USA	Retrospective	41	62.8 ± 12.9	NR	Stage II 11 (26.8) Stage III 28 (68.3) Stage IV 2 (4.9)	Rectal prolapse 41 (100)	MR defecography	NR	NR	NR
Campagna, 2020	Italy	Retrospective	98	60 (44–81)	39 (40.4)	Stage III 60 (61.5) Stage IV 38 (38.5)	Enterocele 53 (53.8) Rectocele 98 (100) Rectal prolapse 98 (100)	Defecography	68 (69.2)	36 (36.5)	Wexner constipation 14 (12–17)
Jallad, 2017	USA	Retrospective	59	55.7 (16.5)	33 (55.9)	≥ II	Rectal prolapse 59 (100)	NR	NR	NR	NR
van Iersel, 2016	Netherland	Prospective	51	56.2 (33.9–75.0)	21 (41.2)	NR	Posterior compartment prolapse 51 (100)	MR defecography and/or an entero-colpo-cysto-defecography	30 (58.8)	33 (64.7)	NR

*MR* magnetic resonance, *NR* not reported, *PFDI* Pelvic Floor Disability Index, *SCP* sacrocolpopexy, *SHP* sacrohysteropexy, *VRP* ventralrectopexy

**Table 3:** Surgical and postoperative characteristics of the studies

Authors and year	Type of surgery, *n* (%)	Concomitant hysterectomy	Postoperative POP stage/anatomic recurrence	Postoperative bulge symptoms	Postoperative rectal prolapse	Postoperative constipation	Postoperative anal incontinence	Subjective assessment of anorectal symptoms	Follow-up (months)
Ross 2024	R-SCP/SHP + VRP	25 (37.3)	Recurrence 5 (7.5) Anterior 3 (4.5) Apical 0 (0) Posterior 3 (4.5)	10 (14.9)	7 (10.4)	14 (20.9)	NR	PFDI 95.7 ± 53.7	≤ 24 *n* = 43 > 24 *n* = 24
Grinstein 2022	L-SHP + VRP: 66 L-SCP + VRP: 19	SCP + VRP: 1/19 (5.3)	NR	8 (9.4)	NR	29 (34.1)	18 (21.2)	Wexner incontinence SHP + VRP 1.1 ± 2.1 SCP + VRP 1.1 ± 1.2 PFDI SHP + VRP 13.4 ± 16 SCP + VRP 6.4 ± 6	SHP + VRP: 24 (4–174) SCP + VRP: 36 (4–174)
Baracy 2021	R-SCP + VRP: 41	14 (34)	Stage II 4/40 (10)	NR	NR	NR	NR	NR	3
Campagna 2020	L-SCP + VRP	59 (59.6)	Stage II 1 (1.9)	0 (0)	NR	30 (30.8)	0 (0)	Wexner constipation 1 (1–3)	12
Jallad 2017	R-SCP/SHP + VRP: 53 (83.8) L-SCP/SHP + VRP: 5 (8.5) O-SCP/SHP + VRP: 1 (1.7)	15 (25.4)	≥ Stage II 5 (8.5)	3 (5.1)	8 (13.5)	NR	NR	NR	17 (1–76)
van Iersel 2016	R-SCP + VRP	30 (58.8)	Distal rectocele 1 (2) Cystocele stage III 1 (2)	NR	NR	8 (26.7)	14 (27.5)	Wexner incontinence score 3.0 (0–17.0) Wexner constipation score 6.0 (0–21.0)	12.5

*BMI* body mass index, *CS* cesarian section, *MUI* mixed urinary incontinence, *MUS* mid-urethral sling, *NR* not reported, *OASIS* obstetrics anal sphincter injuries, *RP* retropubic, *SIS* single incision sling, *SUI* stress urinary incontinence, *TVT* tension-free vaginal tape, *TOT* transobturator tape, *UI* urinary incontinence, *UDI* urogenital distress inventory, *UUI* urgency urinary incontinence, *VD* vaginal delivery

### Results of synthesis

**Fig. 3:**
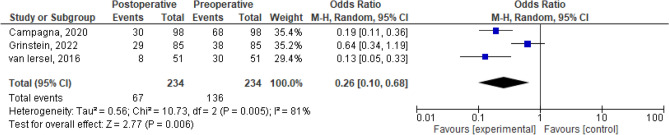
Pooled results of postoperative constipation in women submitted to sacrocolpopexy with ventral rectopexy from a meta-analysis of cohort studies

**Fig. 4:**
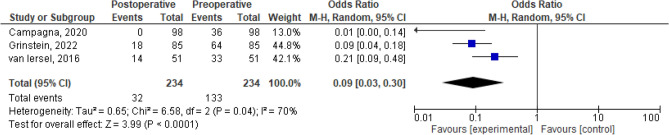
Pooled results of postoperative anal incontinence in women submitted to sacrocolpopexy with ventral rectopexy from a meta-analysis of cohort studies

**Fig. 5:**
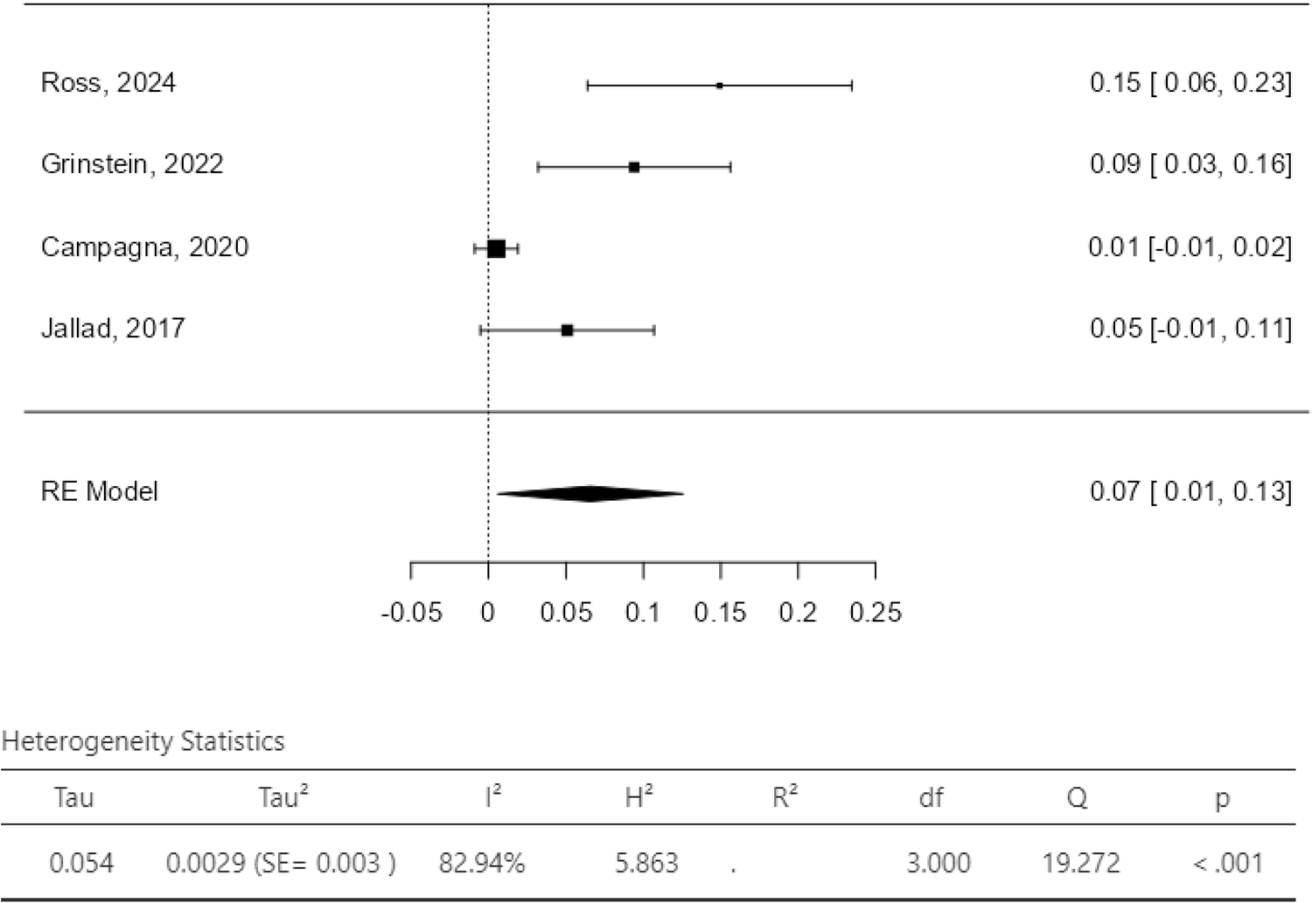
POP subjective relapse (proportion meta-analysis plot—random effect)

**Fig. 6:**
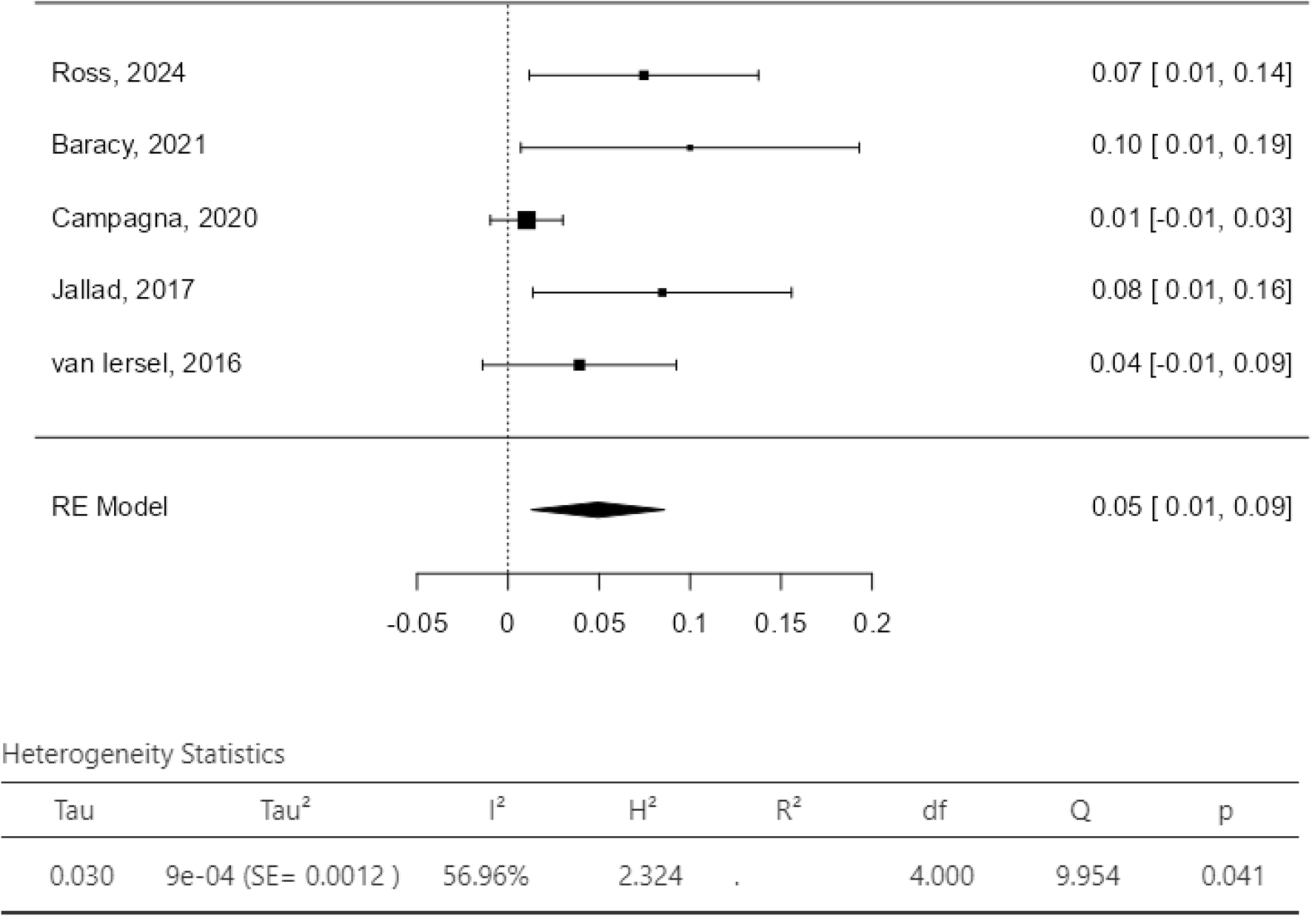
POP objective relapse (proportion meta-analysis plot—random effect)

The dichotomous meta-analysis showed a postoperative improvement of constipation/ODS [OR 0.26, 95% CI 0.10–0.68; *p* = 0.006 (*I*^2^ test 81%, *p* = 0.56); Fig. 3] and of AI/FI [OR 0.09, 95% CI 0.03–0.30; *p* < 0.0001 (*I*^2^ test 70%, *p* = 0.04); Fig. 4] after combined VRP and SCP/SHP. The proportion meta-analysis of the four included studies reported a subjective POP recurrence rate of 7% (95% CI 1–13%; *I*^2^ test 82.9%, *p* < 0.001; Fig. 5). The proportion meta-analysis of the five included studies for objective POP recurrence was 5% (95% CI 1–9%; *I*^2^ test 56.9%, *p* = 0.041; Fig. 6).

Adverse events and reoperation rates are reported in Table 4. No serious adverse events were reported.

**Table 4:** Complications during and after surgery and reoperation reported in the included studies

Authors and year	Peri-operative complication	Mesh complication	Reoperation for POP relapse	Reoperation for posterior prolapse/rectal prolapse relapse	Other reoperation
Ross 2024	Bowel injury 2 (2.9)	0 (0)	NR	NR	NR
Grinstein 2022	Hemorrhage 1/85 (1.2), bladder injury 3/85 (3.6), surgical site infection 1/85 (1.2), postoperative hemorrhage 1/85 (1.2), thrombophlebitis 1/85 (1.2), sepsis 1/85 (1.2)	0 (0)	0 (0)	NR	SUI: 10/85 (11.8)
Baracy 2021	Bowel/mesenteric injury 1 (2.4)	0 (0)	1 (3)	0 (0)	4 (10)
Campagna 2020	0 (0)	0 (0)	0 (0)	0 (0)	0 (0)
Jallad 2017	Conversion to laparotomy 1 (1.7), bladder injury 1 (1.7), wound infection 4 (6.8), ileus 4 (6.8), small bowel obstruction 2 (3.4), pelvic abscess 1 (1.7), sepsis 1 (1.7), DVT/PE 2 (3.4), cardiopulmonary 3 (5.1), discitis 1 (1.7)	Vaginal erosion 1 (1.7)	2 (3.3)	6 (10.1)	Mesh removal for discitis 1 (1.7)
van Iersel 2016	UTI 1 (2), hematoma 1 (2), abscess 1 (2), anterior cutaneous nerve entrapment 2 (3.9), perforating vaginal suture 1 (2)	Vaginal erosion 2 (4)	0 (0)	0 (0)	Partial mesh removal 2 (4), neurectomy 1 (2), removal of the perforating suture 1 (2), transanal mucosectomy 1 (2)

*DVT/PE* deep venous thrombose/pulmonary emboly, *POP* pelvic organ prolapse, *SUI* stress urinary incontinence, *UTI* urinary tract infection

Follow-up of the included studies ranged between 3 and 36 months.

## Discussion

Symptoms of pelvic floor dysfunction symptoms are often associated with multicompartmental POP [12, 13]. Indeed, literature reports a high prevalence of concomitant rectal intussusception (55%), rectal prolapse (38%), rectocele (53%), and enterocele (14%) in patients with urogynecological symptoms [29, 30]. Therefore, a multidisciplinary approach to women with pelvic floor disabilities (PFDs) has become essential to provide the best treatment, and, if surgery is indicated, the optimal type of surgery.

This meta-analysis evaluated the anatomical and functional outcomes of combined VRP and SCP/SHP for the treatment of multicompartmental POP. The results showed significant postoperative improvements in constipation/ODS and AI/FI symptoms, along with a low subjective and objective pelvic organ prolapse recurrence rate of 7 and 5%, respectively. The low rates of recurrence, and absence of severe adverse events, demonstrated the safety and efficacy of this combined approach. As reported in D’Hoore’s description of the ventral rectopexy technique, the posterior vaginal fornix was elevated and sutured to the anterior aspect of the rectum to allow correction of vaginal vault prolapse [16]. This kind of surgery allows a excellent posterior and apical anatomical result, in addition to the sacrocolpo/hysteropexy procedure. In addition, the anorectal functional results of this meta-analysis are encouraging. Indeed, the rates of ODS and AI/FI symptoms were highly improved postoperatively. This could be related not only to the restoration of the anatomy but also to the procedure, which avoids any posterolateral dissection of the rectum, which could lead to the injury of the autonomic nerves and, consequently, to dysmotility and impaired evacuation. The reported excellent results in terms of bowel and anal function in included patients may be explained by the fact that the described surgical technique avoids posterolateral dissection of the rectum.

The 2016 study by Van Iersel et al. showed significant success with the robotic-assisted combined VRP and SCP. Improvements in patients’ quality of life were evident, with significant reductions in constipation (73.3%) and fecal incontinence (57.6%). A particularly noteworthy aspect of this study was the improvement in sexual function, with a significant increase in the PISQ-12 score, suggesting that the procedure not only enhanced pelvic functions but also positively impacted sexual quality of life. However, the study had limitations, including a 1-year follow-up period and the absence of a control group, which limited the generalizability of the findings. Nevertheless, the results suggested that robotic surgery offered advantages, particularly in terms of precision, reduced operative times, and improved visualization [18].

A study published in 2017 by Jallad et al. analyzed the combined ventral rectopexy and sacrocolpopexy (or hysterocolpopexy) procedure in women with POP. The results showed significant improvements in both anatomical and functional outcomes, with high patient satisfaction and low rates of reoperation. However, a number of postoperative adverse events were reported, particularly ileus, small bowel obstruction, and wound infection. An interesting aspect of the study was the use of biological grafts to suspend the rectum, which was associated with a higher rate of postoperative complications. Although generally preferred over synthetic mesh owing to a lower risk of long-term mesh erosion, biologic grafts appeared to increase the likelihood of adverse events, particularly in patients undergoing extensive dissection or those with significant comorbidities. The study also reported a rare but serious complication of discitis, suggesting that grafts should be secured at or below S1 to prevent this issue. Major limitations of this study included its retrospective design and the lack of long-term follow-up [19].

The study by Campagna et al. explored the laparoscopic approach for the combined treatment of POP and RP. Multicompartmental assessment was considered crucial for planning an effective surgical intervention, allowing for the treatment of all prolapses in a single procedure and minimizing the need for subsequent surgeries. The combined treatment resulted in favorable anatomical and subjective outcomes, with significant improvements in patients’ quality of life. In particular, the risk of mesh erosion was reduced owing to the abdominal approach, which avoided the vaginal route and minimized the risk of postoperative infection or mesh erosion. Sexual function also improved, with no adverse effects in patients who underwent supracervical hysterectomy. However, the authors emphasized the need for long-term studies with control groups to confirm the promising results observed in the short- and medium-term results [20].

Baracy et al. in 2021 aimed to determine whether concomitant ventral mesh rectopexy during robot-assisted sacrocolpopexy (R-SCP) reduced the rate of subsequent posterior vaginal wall prolapse compared with sacrocolpopexy alone. The results showed an improvement in posterior vaginal wall integrity on POP-Q assessment, with patients who underwent R-SCP with concomitant ventral mesh rectopexy requiring less posterior wall corrective surgery. Although the data suggested a trend towards a reduction in subsequent posterior wall prolapse, this difference was not statistically significant owing to an insufficient sample size. This study, which was the first to investigate this issue, had limitations related to its retrospective design, lack of long-term follow-up, and a lack of sufficient statistical power to detect significant differences [21].

Grinstein et al. in 2022 evaluated the safety of a combined laparoscopic procedure to correct POP and RP with mesh placement. The results showed a significant improvement in anal incontinence, with an increase in the WFI score. However, a rare complication of spondylodiscitis was observed in one patient, although it was difficult to attribute this directly to the VRP procedure. The main limitation of this study was its retrospective design, with difficulties in accurately determining the timing of recurrence owing to a median follow-up period of only 24 months. Nonetheless, the study’s strength lay in the relatively large number of patients treated by an experienced surgeon, with standardized pre- and postoperative evaluations [22].

Finally, the 2024 study by Ross et al. assessed patient-reported functional outcomes in individuals who underwent robot-assisted ventral rectopexy and sacrocolpopexy for combined prolapse. Patients reported low levels of symptom bother related to pelvic floor disorders, with improvements in all PFDI-20 subscales except for the UDI-6. Sexual function was notably high after surgery, with patients reporting an overall sense of improvement. The complication rate of the combined procedure was minimal, and recurrence rates for both rectal and pelvic organ prolapse were low. However, the study had several limitations, including missing preoperative PFDI-20 data for some patients, making it difficult to fully assess symptom improvement, and the lack of a control group for comparison with isolated procedures. Despite these limitations, the study provided valuable data on the safety and efficacy of the combined robot-assisted procedure [23].

The results of our meta-analysis confirmed that the combined VRP and SCP/SHP procedure was a promising therapeutic strategy for patients with multicompartmental pelvic organ prolapse with posterior pelvic floor disorders. The significant improvement in symptoms of constipation and fecal incontinence, as assessed by postoperative outcome measures, suggests that the surgical approach not only restores anatomy but also contributes to enhanced quality of life for patients. The low rates of both subjective and objective prolapse recurrence support the notion that the combination of these two techniques provides durable results.

Several limitations are present in this study. The scarcity of randomized controlled trials, combined with the predominance of retrospective studies, inherently introduces methodological limitations. The analysis showed some heterogeneity between the included studies, as indicated by the *I*^2^ heterogeneity indices for different outcomes, suggesting that further high-quality studies are needed to confirm and generalize these findings. Differences in study design, sample size, and, in some cases, relatively short follow-up periods may affect the robustness of the results. Despite these limitations, this meta-analysis possesses several strengths. Even if individual study sample sizes are small, the included population is of significant clinical importance for healthcare providers confronted with multicompartment prolapse. Another aspect that deserves attention is the accuracy of diagnostic techniques, such as defecography, in identifying posterior compartment defects, which is crucial for the correct surgical indication. Although the combined procedure showed a low incidence of serious adverse events, it is essential to monitor patients closely over the long term, as some complications might occur beyond the follow-up period considered in this study.

## Conclusions

Ventral rectopexy associated with sacrocolpo/hysteropexy was demonstrated to be safe and effective for women affected by multicompartment pelvic organ prolapse, providing optimal functional and anatomical results. Larger, long-term, prospective controlled studies are necessary to confirm these results.

## Data Availability

No datasets were generated or analyzed during the current study.
